# Reconstructive Surgery and Joint-Sparing Surgery in Valgus and Varus Ankle Deformities: A Comprehensive Review

**DOI:** 10.3390/jcm11185288

**Published:** 2022-09-08

**Authors:** Silvio Caravelli, Giulia Puccetti, Emanuele Vocale, Marco Di Ponte, Camilla Pungetti, Annalisa Baiardi, Alberto Grassi, Massimiliano Mosca

**Affiliations:** 1 II Clinic of Orthopaedics and Traumatology, IRCCS Istituto Ortopedico Rizzoli, 40136 Bologna, Italy; 2 Department Orthopaedics and Traumatology, Ospedale Maggiore “Pizzardi”, 40133 Bologna, Italy

**Keywords:** ankle deformity, early osteoarthritis, reconstruction, joint-sparing, surgery, review

## Abstract

Osteoarthritis (OA) of the ankle affects about 1% of the world’s adult population, causing an important impact on patient lives and health systems. Most patients with ankle OA can show an asymmetrical wear pattern with a predominant degeneration of the medial or the lateral portion of the joint. To avoid more invasive ankle joint sacrificing procedures, joint realignment surgery has been developed to restore the anatomy of the joints with asymmetric early OA and to improve the joint biomechanics and symptoms of the patients. This narrative, comprehensive, all-embracing review of the literature has the aim to describe the current concepts of joint preserving and reconstructive surgery in the treatment of the valgus and varus ankle early OA, through an original iconography and clear indications and technical notes.

## 1. Introduction

Ankle osteoarthritis (OA) affects about 1% of the world’s adult population [1], causing an important socioeconomic impact on patients and health systems [2,3]. Unlike the hip and the knee, ankle OA is reported in about 80% of the cases related to trauma [4,5], and a part of the population involved is represented by active and high-performance-demanding patients, with a large part younger than 50 years [1].

Most patients with ankle OA show an asymmetrical wear pattern [6] with a predominant degeneration of the medial or the lateral portion of the joint. If left untreated, these patients will have a progression of the disease up to end-stage global osteoarthritis [7].

To avoid more invasive ankle joint sacrificing procedures, joint realignment surgery has been developed to restore the anatomy of the joints with asymmetric early osteoarthritis and improve the biomechanics of the joints and symptoms of the patients [6,7,8]. This narrative, comprehensive, all-embracing review of the literature has the aim to describe the current joint preserving and reconstructive surgery in the treatment of the valgus and varus ankle early osteoarthritis.

## 2. Etiology

About 80% of ankle OA has a post-traumatic origin, such as articular fractures or repeated trauma [9,10]. The fractures that can lead to an asymmetric *valgus* ankle are represented by the distal diaphysis of the tibia, tibial plafond, and distal fibular fractures [3]. Another frequent cause of *valgus* ankle osteoarthritis is repeated ankle sprains with associated insufficiency of the deltoid ligament complex [11].

The deltoid ligament complex has a fundamental role in preventing the lateral translation and valgus inclination of the talus [12]. It has been demonstrated that lesions of the deep portion of the deltoid ligament can lead to a lateral translation of the talus and cause valgus ankle osteoarthritis [11,12,13]. Moreover, the adult flatfoot deformity associated with tibialis posterior (TP) dysfunction and hindfoot abnormalities can be recognized as a cause of ankle OA [14,15].

*Varus* deformities can also be related to previous fractures (mostly lower-leg fractures [16,17] and/or repetitive ankle sprains [18]), as well as bone deformities, chronic lateral capsulo-ligamentous insufficiency or muscle imbalance, or neurologic diseases such as cerebral palsy or nerve disorders. As with valgus deformities, the *varus* ones can be associated with a concomitant deformity that may be seen at the supramalleolar, intraarticular, and/or inframalleolar level [18,19,20].

## 3. Management of *Valgus* Ankle Early Osteoarthritis

### 3.1. Diagnosis

#### 3.1.1. Clinical Assessment

It is necessary to obtain a full clinical history of the patient, including comorbidities, previous surgeries, trauma, and fractures. Both the lower limbs need to be fully exposed; it is necessary to evaluate the alignment of the entire limb during weight-bearing and the posture during standing and walking [11].

The painful areas of the ankle and foot have to be evaluated, as well as the motion of the joints. The instability of the medial or lateral ligamentous complex should be clinically tested. Tightness of the Achilles tendon has to be studied with the knee in extension and flexion.

Tiptoe position and the function of the tibialis posterior tendon should be assessed; the hindfoot alignment, plantar arch, and midfoot are then considered. The mobility of the hindfoot and midfoot and any deformity should be highlighted and defined if mobile or rigid [11]. The position of the forefoot, in particular of the first ray, should be determined, as well as the eventual supination or pronation of the forefoot and toe deformities [21].

#### 3.1.2. Radiographic Assessment

In the literature, authors routinely reported weight-bearing standardized radiographs for radiographic evaluation. These require anteroposterior and lateral views of the ankle and lateral and dorsoplantar views of the foot [3,22]. Besides, it is also useful to perform a *mortise view* of the tibiotalar joint [14]. A Saltzman view is included for the evaluation of the hindfoot alignment.

Patients with a deformity involving the ipsilateral hip or knee should be assessed with a panoramic weight-bearing radiograph of the entire limb.

**Figure 1:**
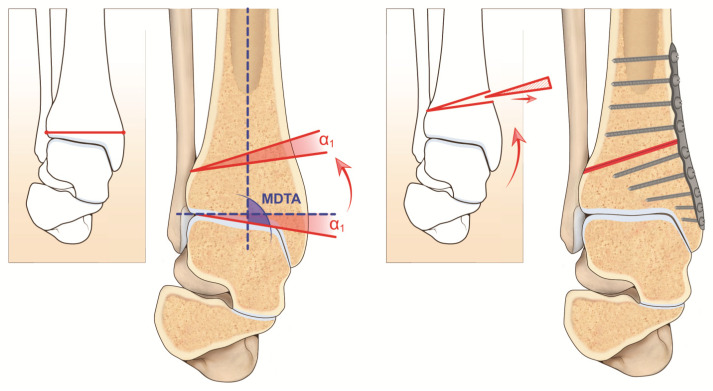
Visual artwork showing α angle of correction and MDTA (medial distal tibial angle). α: angular correction desired.

One of the most important parameters for the analysis of the valgus deformity of the tibiotalar joint is the medial distal tibial angle (MDTA, Figure 1), which measures the angle between the longitudinal axis of the tibia and the transverse axis of the tibial plafond. The mean value reported in the literature differs between measurements on radiographs and studies on cadavers. In vivo radiographic measurements reported a mean value of 92.4 ± 3.1° (84–100°) and studies on cadavers a mean value of 93.3 ± 3.2° [3,23]. Moreover, the measures could differ between radiographs in standard and mortise views of the ankle; it is recommended to perform the angle measurements on standardized radiographs.

Another fundamental measurement for the preoperative evaluation of a valgus ankle is the medial tibiotalar angle (MTTA). The MTTA measure the position of the articular surface of the talus concerning the main axis of the tibia, with a mean value of 9.5° ± 1.2° [14]. The difference between the MDTA and the MTTA gives the talar tilt and measures the incongruence of the tibiotalar joint [4]. The talar tilt in a normal aligned ankle has a value of less than 4° [24]. As reported by Krähenbühl et al. [25], the MDTA and talar tilt are useful measurements to distinguish between a supramalleolar and an intraarticular deformity. An MDTA value greater than 92° indicates a supramalleolar valgus deformity. A talar tilt greater than 4° is related to an incongruent tibiotalar joint and an intraarticular deformity.

In selected cases with degenerative alterations of the ankle and other adjacent joints, it is recommended to use single-photon emission computed tomography (SPECT/CT) to evaluate the exact location of the degenerative joint changes and their biological activity [21,26].

Eventually, magnetic resonance imaging (MRI) will be useful in the evaluation of the amount of residual cartilage layer, the grade of joint degeneration, eventual osteochondral lesions, and the quality of the ankle ligaments and tendons [22,27].

## 4. Indications and Contraindications

The main indication for a realignment surgery procedure is represented by an asymmetric OA with a valgus deformity associated with a lateral articular surface preserved [7,28]. Some authors reported that at least 50% of the articular surface should be preserved for this surgery [4,29]. In selected patients, another indication is isolated lateral osteochondral lesions of the ankle [30]. In some cases, a realignment procedure represents the first surgical step in ankle replacement surgery [31], making it easier and faster. It reduces the time of surgery and complications and improving the final alignment of the definitive components [3].

The contraindications to a realignment surgery can be found in an end-stage OA with less than 50% of cartilage preserved, patients in poor general health or not able to follow the postoperative protocol [28], vascular or neurological diseases, poor bone quality [4], acute or chronic infections of the ankle, unmanageable hindfoot instability [3], or neuropathic disorders.

Relative contraindications are represented by advanced age (older than 70 years) [3], smoking, diabetes mellitus, soft tissue or skin disorders, or abnormalities [4,32].

## 5. Supramalleolar Osteotomy

A supramalleolar osteotomy is a surgical procedure introduced by Takakura et al. [14,33] in 1995. In a *supramalleolar valgus deformity*, the most indicated surgical procedure is a medial closing wedge osteotomy.

### 5.1. Preoperative Planning

To determine the height of the wedge of the osteotomy, the width of the distal tibia (W) is measured on an anteroposterior weight-bearing radiograph. The height of the wedge to remove from the tibia is the result of the formula: H = tan α1 × W, with α1 as the desired angular correction [3,34] (Figure 1). The osteotomy distal plane should be perpendicular to the medial cortex of the tibia. The proximal plane is inclined based on the angle of correction desired and the height of the wedge to remove (as a general rule, it is suggested there be an overcorrection of 2–4°) [4]. For anomalous MDTA, the center of rotation of angular (CORA) is measured. It is represented by the intersection of the mid-diaphyseal line and the line from the center of the joint and perpendicular to the abnormal MDTA. CORA may be at the level of the joint line (usually due to misalignment or degeneration of the anatomical joint line) or proximal (usually due to tibial defects/fractures) [34,35,36].

### 5.2. Clinical Results

Hintermann et al. [37] reported 48 cases of ankle malunited fracture sequaele, treated by supramalleolar osteotomy, finding a correction of malalignment in all patients at the follow-up. At the follow-up of 7.1 years, good–excellent results were found in 42 patients. Pagenstert et al. [7] reported 35 cases of realignment surgery for valgus or varus osteoarthritis. At a mean follow-up of 5 years, the authors found a significant improvement in pain and functionality.

Knupp et al. [8] reported 92 patients with valgus or varus malalignment treated with realignment surgery. At a mean follow-up of 43 months, they reported clinical and radiological improvements, with a subsequent ankle arthrodesis or arthroplasty in 10 patients.

## 6. Distal Fibular Lengthening Osteotomy

The unsatisfactory reduction of a distal fibular fracture, or the loss of the reduction obtained with the surgery, could lead to an alteration of the anatomical axis and distribution of the loads on the articular surfaces, with a consequent deformity in the valgus of the tibiotalar joint [38,39]. The incidence of malunion after ankle fractures is reported as between 5% and 68% [40,41].

The fibula represents the tibiotalar joint lateral buttress and contributes to maintain the normal anatomy and position of the talus in the mortise. The most frequent malunion of the fibula is shortening and external rotation and can lead to a widening of the mortise and lateralization and instability of the talus [42,43]. Typically, an ankle with a malunion of the fibula is characterized by a widened medial joint space due to the external rotation and lateral translation of the talus [43,44,45]. As described, the translation and instability of the talus have important implications for the function of the tibiotalar joint: a translation of 1 mm of the talus can lead to a reduction of the joint contact surface by up to 40% [38,39,44].

**Figure 2:**
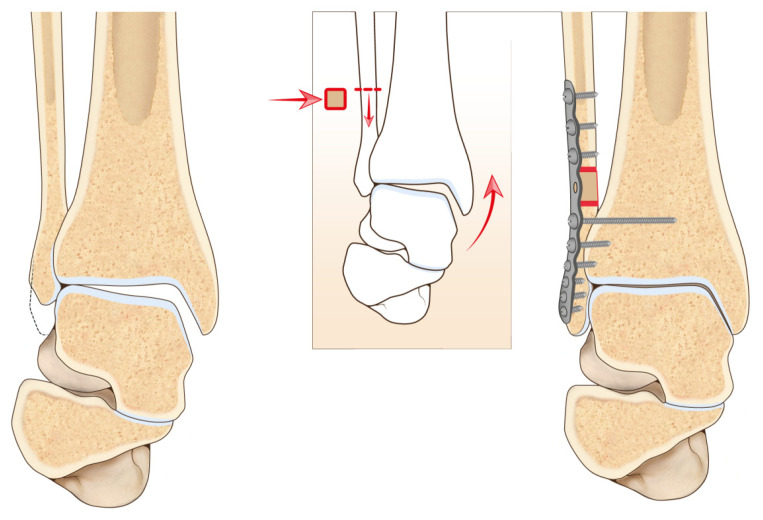
Visual artwork showing fibular lengthening osteotomy technique.

Speed et al. [41] first described the corrective osteotomy of a malunited fibula in 1936. Since then, several authors have reported encouraging results with this type of surgical procedure. Weber et al. [46] described a Z-lengthening osteotomy, Roberts et al. [47] described an oblique osteotomy at the level of the initial fracture, and Yablon and Leach [48] used a transverse fibular osteotomy with or without the use of bone graft (Figure 2).

The oblique or Z-lengthening osteotomy is indicated only for shortening of minimum entity and malrotations of less than 10°. In cases of important deformities, a transverse osteotomy must be preferred [49]. It is important to note that in this procedure the debridement of the syndesmotic scar tissue is mandatory to enable lengthening the fibula, otherwise, the fibula cannot be pushed downwards to the tibiofibular joint [46]. Due to the altered anatomy of the fibula and also due to instability of the syndesmosis, it is common to detect a high talar tilt value on the radiograph. Most authors measured radiographic parameters on weight-bearing radiographs internally rotated at 20° [43].

Three main alterations of the normal anatomy of the ankle are common on an anteroposterior 20° internally rotated radiograph: unequal joint space, a broken Shenton’s line, and a broken curve between the lateral part of the talar articular surface and fibular recess [38,43,50]. Other parameters important for the evaluation and planning are the talar tilt, the talocrural angle, and the bimalleolar angle.

**Figure 3:**
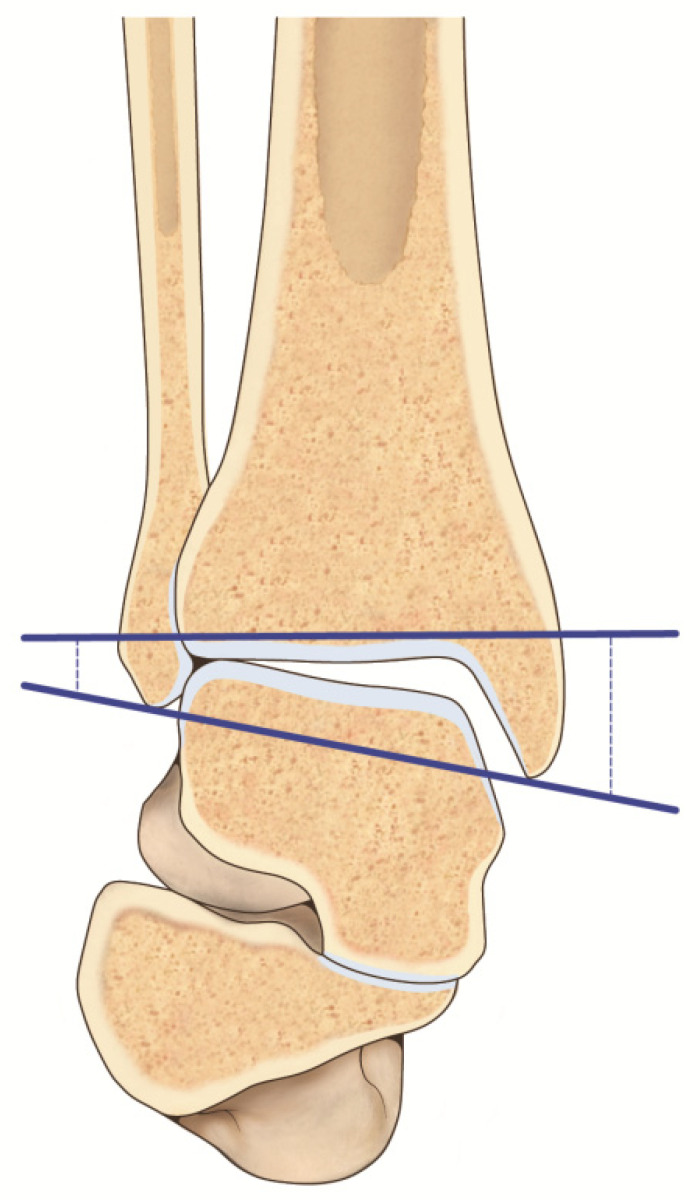
Visual artwork highlighting the bimalleolar angle.

Leaving out the already explained talar tilt, the talocrural angle represents the angle between the line of the tibial plafond and a line connecting the tips of the lateral and medial malleolus [43]. If this measurement is greater than 3° compared to the contralateral side, a fibular shortening is present. The bimalleolar angle is formed by a line connecting the tips of the two malleoli and a vertical line that follows the fibular intramedullary space (Figure 3). A difference greater than 2.5° compared to the contralateral side indicates a fibular shortening [43,51].

To assess the amount of fibular shortening on the affected side, it can be useful to compare the relationship between the medial and lateral malleolus to those on the normal contralateral ankle [42].

Computerized tomography (CT) is useful for the evaluation of malrotation and to detect the incongruence of the lateral malleolus in the fibularis tibiae notch [42,46].

### Clinical Results

Weber et al. reported 23 cases of fibular malunion treated with lengthening osteotomy, with 73% good–excellent results [50]. El-Rosasy et al. [42] described 17 cases, with 12 good or excellent and 5 unsatisfactory results. Van Wensen et al. [43] found, in their case series, 75% good or excellent results. Mosca et al. [38] reported the outcomes of 23 patients treated with ankle joint rebalancing through fibular lengthening, with an improvement of the AOFAS score from a preoperative 32.6 ± 7.6 points to a postoperative score of 74.0 ± 8.9 points and all cases treated with radiographic evidence of good alignment.

## 7. Ligament Reconstructions

The deltoid ligament complex has a fundamental role in the medial stability of the ankle. Besides, it contributes to preventing the lateral translation and the valgus angulation of the talus [11,12]. In case of concomitant insufficiency of the deltoid ligament, its reconstruction is indicated.

As reported by Hogan et al. [11], the reconstruction of the deltoid ligament complex represents a valid additional treatment when ligamentous stability and balancing are required. Instead, an isolated reconstruction of the deltoid ligament is not an effective treatment of the valgus ankle OA and cannot have satisfactory outcomes as an isolated procedure.

Various techniques have been described for the repair or reconstruction of the deltoid ligament. It is possible to repair the deltoid ligament anatomically by reattachment and retensioning it to its origin on the medial malleolus with anchors or trans-osseous sutures [14]. Wiltberger and Mallory described a technique using a tendinous autograft, splitting the posterior tibial tendon (PTT). The PTT was left attached to its insertion and the split proximal end passed through a bone tunnel in the medial malleolus [11,52].

## 8. Associated Procedures (Calcaneal Osteotomy, Evans Osteotomy, Subtalar and Midfoot Arthrodesis, Cotton Osteotomy, Posterior Tibial Tendon Repair or Reconstruction)

In cases of associated valgus deformity of the hindfoot or of the mid- or forefoot, or posterior tibial tendon dysfunction, additional procedures could be considered.

## 9. Management of Varus Ankle Early Osteoarthritis

### 9.1. Diagnosis

#### 9.1.1. Clinical Assessment

The patient’s examination has a central role in the evaluation work-up: during the clinical examination, it is important to expose not only the foot but the entire lower limb because the varus malalignment can be related not only to the ankle but also to the entire lower limb. The patient must be examined barefoot, during walking and standing, and the position of the ankle, the foot, and the hindfoot should be assessed. It is not rare to point out heel varus, cavus foot, and/or a first ray plantarflexion while examining the foot during weight-bearing. Using the Coleman Block test, it is possible to assess the role of the first ray on the varus hindfoot position. Clinical evaluation should also take into consideration the compensatory valgus position of the hindfoot. After visual examination, palpation must be performed; it is important to focus on the search for a tender spot on the course of the medial and lateral ligament complexes and tendons; the joint lines of the ankle, subtalar, and Chopart joints should be always palpated to highlight painful points.

The functionality of muscles should be evaluated and particular attention must be paid to possible tightness of the heel cord and function of plantar flexors. The stability of the ankle and the hindfoot should be manually assessed. It is also important to measure the tibiotalar range of motion both in plantarflexion/dorsiflexion and in eversion/inversion.

The last part of the clinical examination should include a neurovascular status examination and in particular tibial nerve function. 

#### 9.1.2. Radiographic Assessment

The radiographic examination is performed through weight-bearing X-rays: dorso-plantar and lateral plain radiographs of the foot, ankle mortise, and hindfoot alignment view (Saltzman view).

Panoramic lower limb radiographs should also be included allowing the surgeon to assess the bony deformities of the entire lower extremity. Preoperative CT scans, SPECT-CT [26], or a weight-bearing CT (WBCT) could be useful to better comprehend the case, assess the quality of the bone, and identify cysts preoperatively [53]. Magnetic resonance imaging (MRI) may be helpful to evaluate possible tendon and muscle pathologies [54].

#### 9.1.3. Surgical Treatment

If conservative treatment has given no benefit to the patient, surgical treatment should be taken into consideration. The surgical options are joint-sparing techniques, arthrodesis, and arthroplasty. Regardless of the technique, the goal is to reach a plantigrade, fully functional, and stable foot. To obtain an optimal treatment, associated deformities, forefoot malalignment, lesser toe deformities, Achilles tendon tightness, knee deformities, and contralateral lower limb malalignment, lateral chronic ankle instability, and hindfoot OA must be recognized.

## 10. Supramalleolar Osteotomy

**Figure 4:**
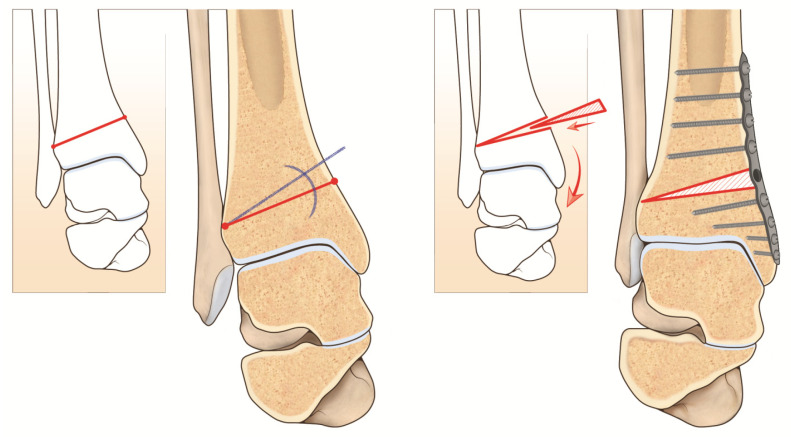
Visual artwork showing supramalleolar osteotomy in varus ankle deformity.

The aims of supramalleolar osteotomy (SMOT) are both restoration of the lower-leg axis (to improve intra-articular load distribution) and to slow down or to stop the degeneration of the tibiotalar joint [3,55,56] (Figure 4).

Before performing a SMOT it is important to assess different parameters preoperatively, such as the medial distal tibial angle to quantify the supramalleolar varus deformity, the tibiotalar tilt to evaluate intra-articular deformity in the coronal plane, and the calcaneal moment arm to quantify inframalleolar deformity in the coronal plane. Based on leg length, the osteotomy can be performed either in a medial opening-wedge or lateral closing-wedge fashion when the varus deformity is larger than 10° and/or a previously fused distal tibiofibular joint is present [16]. It may (in most cases) or may not be associated with a fibular osteotomy in case of an overlong fibula. Knupp and colleagues [34] modified a classification of varus ankle providing a treatment algorithm for the joint sparing procedure. It is extremely useful if an isolated supramalleolar osteotomy may not be sufficient to correct the deformity.

Depending on the case, different procedures could be associated, such as an intra-articular distal tibia osteotomy, if varus talar tilt persists due to asymmetric joint wear (it could be done simultaneously with medial opening wedge osteotomy as described by Hintermann and colleagues [57]). Other concomitant procedures could be done if ligamentous instability or fixed hindfoot deformity is present [7].

### Indications and Contraindications

The common indications for SMOT are represented by supramalleolar varus deformity with a partially preserved tibiotalar joint, end-stage ankle OA requiring total ankle replacement, or ankle arthrodesis (as a staged procedure to improve the overall leg axis).

Contraindications to SMOT are end-stage varus OA with degeneration of the entire tibiotalar joint.

## 11. Discussion

Ankle OA results from mainly post-traumatic causes. The typical patient is relatively young and active and because of that, their expectations are higher than those of the patient affected by hip or knee OA. In the early stages of varus ankle OA, the best option is represented by an osteotomy to shift the weight load from medial to lateral [3,55,56]. Through surgery, it is possible to resume normal biomechanics and to achieve pain relief, functional improvement, and also slow down the degeneration process [7,33,58,59,60,61,62].

On the other hand, it is important to underline that 25% of patients who undergo a SMOT procedure need a secondary procedure [63]. Krähenbühl and colleagues [6], in a prospective study showed the need of a secondary ankle replacement or an ankle arthrodesis at long-term follow-up.

Risk factors associated with failure of an early OA treatment are young age at the time of the surgery and a Takakura preoperative score 3B. Additionally, a preoperative tibiotalar varus tilt greater than 7° can be considered a risk factor [6], as well as a varus ankle with the talus tilted within the mortise, and degenerative changes located in the medial gutter as described by Tanaka [64].

On the other hand, Kim and colleagues [55] in their retrospective study showed significant pain relief in most of the 31 patients who underwent supramalleolar medial wedge OT (Visual Analog Scale (VAS) 7.1 + 0.8/3.4 + 1.3); they also showed a functional improvement (American Orthopedic Foot and Ankle Society (AOFAS) hindfoot score 62.9 + 4.0/83.1 + 7.5) at a mean of 13.2 + 1.4 months after surgery. In another series of 35 patients who had undergone supramalleolar osteotomies by Pagenstert and colleagues [7], 91% postponed total ankle replacement.

Today, computer tomography-based 3D planning allows the operators to make a precise prediction of the correction and the possibility of performing the surgical procedure using patient-specific cutting guides, increasing its accuracy [65].

However, it is important to underline the fact that complete pain relief cannot be achieved by SMOT because of preexisting irreversible degenerative changes of the tibiotalar joint.

## 12. Complications and Pitfalls

The intraoperative complications of these procedures include neurovascular or tendon lesions. An accurate surgery is necessary to minimize this risk [3].

Wound healing problems are frequent, above all in patients with risk factors such as smokers, diabetes, or poor quality of soft tissues.

Over- or under-correction of the deformity can be linked to inaccurate preoperative planning or technical errors [48] and could lead to an impingement syndrome, deformity of the ankle, or faster cartilage wear. 

A malunion or nonunion can occur at the site of the osteotomy. Among the possible causes of this complication, inappropriate surgical technique, violation of the lateral cortex of the tibia [3], and a non-anatomic reduction or a secondary displacement are included.

A loss of correction is possible in case of non-compliance of the patient regarding the early postoperative period or case of inadequate fixation [14].

The bone graft used in the procedure of fibular lengthening could be reabsorbed or collapse. An appropriate surgical technique with a stable fixation could prevent this complication [48].

Moreover, pain or discomfort at the level of the hardware is possible, and in some cases, a subsequent hardware removal procedure after the consolidation of the osteotomy is necessary.

Months after the procedure, a progression of osteoarthritis of the tibiotalar joint can occur [6].

One of the most feared complications is infection, which can be superficial or deep [66]. A superficial infection can appear as delayed or poor healing of the wound, necrosis of the wound, or hematoma. In contrast, deep infection can involve hardware, soft tissues, or reach the bone.

## 13. Summary

Patients with osteoarthritis of the ankle in most cases present a varus or valgus malalignment.

When osteoarthritis has not reached its final stage, a joint realignment surgical procedure can slow down the cartilage wear and relieve symptoms. Realignment surgery of the ankle has to be carefully planned, evaluating the origin of the deformity and associated pathological conditions of the ankle and the foot.

The literature has shown promising results for the various techniques available for the correction of ankle malalignment.
